# Reducing sugar and aroma in a confectionery gel without compromising flavour through addition of air inclusions

**DOI:** 10.1016/j.foodchem.2021.129579

**Published:** 2021-08-30

**Authors:** Katy Su, Daniel Festring, Charfedinne Ayed, Qian Yang, Craig J. Sturrock, Robert Linforth, Tim Foster, Ian Fisk

**Affiliations:** a The University of Nottingham, Division of Food, Nutrition and Dietetics, School of Biosciences, Sutton Bonington Campus, Loughborough LE12 5RD, UK; b The University of Nottingham, Agricultural and Environmental Sciences, Sutton Bonington Campus, Loughborough LE12 5RD, UK; c Nestlé Product Technology Centre Confectionery,P.O. Box 204, Haxby Road, York YO91 1XY, UK; d The University of Adelaide, North Terrace, Adelaide, South Australia, Australia

**Keywords:** Aeration, Flavour release, Flavour perception, Calorie reduction

## Abstract

Sugar plays an important role in both the flavour and structure of confectionery. Targets have been set to reduce sugar; however, common strategies often result in changes in flavour and consumer rejection. In this study, an approach was developed to reduce sugar in confectionery gels by aeration, without significantly affecting perceived chewiness. Gelatine based gels with a 23% and 38% reduction in density were formulated using aeration. Mean bubble size was consistent across all gels (0.05–0.06 mm). Time-intensity sensory evaluation was carried out by a trained sensory panel (n = 10). With aeration, no significant difference in overall flavour perception was observed in the 23% and 38% reduced sugar and aroma gels. Air inclusions create a greater surface area, therefore accelerating mass transfer of volatiles and release of sucrose at the food-saliva interface. Consequently, we propose that less calorie dense products can be produced without compromising flavour by using gel aeration technologies.

## Highlights

• Slight aeration of confectionery gels does not impact chewiness perception.
• Air as a low calorie inclusion in gels enhances sweetness perception over time.
• Reduction of aroma, sugar and calories in gels without affecting flavour perception.
• X-ray computed tomography to measure bubble size and distribution.

## 1 Introduction

A high intake of dietary sugars is considered to be associated with increased risks of many health problems such as type two diabetes (Macdonald, 2016). Whilst these impacts are well known, reducing per capital sugar consumption to below recommended levels is still an ongoing challenge in most parts of the world. Conventional strategies to reduce sugar in foods, such as the use of sweeteners, often lead to a change in flavour perception and subsequently consumer rejection of the reformulated product. For example, sugar substitutes may have bitter tastes in addition to the desired sweetness and in some subgroups of the population, consumption to excess may lead to other health issues, such as gastrointestinal problems (Kroger, Meister, & Kava, 2006). The functionality of sugar in foods extends beyond flavour, for example it is also important for overall texture, management of water activity and helps preserve products and may help to extend shelf life (Burey, Bhandari, Rutgers, Halley, & Torley, 2009).

Rather than substituting sugar, the addition of low-calorie inclusions to food products may be an alternative strategy to improve nutritional value, without compromising flavour. Low-calorie inclusions, such as air, can act as fillers that occupy volume in gels (Goh, Leroux, Groeneschild, & Busch, 2010), thereby reducing product density and hence reducing calories per unit volume of food (Zúñiga & Aguilera, 2008), making it more favourable for consumers. The potential of gas inclusions has previously been demonstrated using soft, semi-solid gels aerated to different levels. Goh et al. (2010) showed that with increased aeration of the gels, sweetness or saltiness perception could be maintained when tastant concentrations were decreased and that taste perception increased when tastant levels were kept the same. Similarly Chiu, Hewson, Yang, Linforth, and Fisk (2015) showed that increased aeration of a soft gel resulted in greater saltiness perception and increased both maximum intensity of release and total aroma release of 1-octen-3-ol. However it should be noted that in both these studies, perception was measured as a static response rather than measuring the changes in perception that occur over the time course of consumption. Furthermore, aromas are present in real food systems as mixtures; therefore, it is important to understand the release of a range of aroma compounds with different chemical properties to best tailor an optimal blend during product reformulation.

Although the effect of air inclusions on sensory perception from soft gels has been shown, there is less evidence of how it affects aroma release. The effect of air inclusions on aroma release has been better studied in other model systems, for example liquid foams (Minor, Vingerhoeds, Zoet, Wijk, & Aken, 2009) and egg-albumen foams (Tyapkova, Siefarth, Schweiggert-Weisz, Beauchamp, Buettner, & Bader-Mittermaier, 2016). The effect of aeration and carbonation on flavour release has not only been shown in foods but also beverages, where gas bubble formation and release in beer increased volatile compound release (Clark, Linforth, Bealin-Kelly, & Hort, 2011). In a study focussing on instant coffee containing gas inclusions, a faster release of individual aroma compounds into the headspace was observed, this release was dependent on the physicochemical properties of the individual aroma compounds (Yu, Macnaughtan, Boyer, Linforth, Dinsdale, & Fisk, 2012). Hewson, Hollowood, Chandra, and Hort (2009) evidenced the influence of gustatory, olfactory and trigeminal interactions on sensory perception in a model carbonated beverage, highlighting the differential effects of glucose and fructose on key sensory attributes.

Gas inclusions in foods also significantly affects the texture of the product, for example a softer texture was perceived in chocolate with CO_2_ gas inclusions (Haedelt, Beckett, & Niranjan, 2007). Important textural attributes for gel confectionery include hardness and chewiness, where hardness describes the texture of the first bite and chewiness describes the texture perceived during chewing (Brandt, Skinner, & Coleman, 1963). Changes in texture and appearance upon aeration of a product has been discussed in previous studies (Chiu et al., 2015, Goh et al., 2010). As well as reducing the energy intake from snacks by lowering the calorie density of products, a study also showed the degree of aeration of food affects the volume consumed, and satiety was increased when the more aerated samples were presented (Arboleya, García-Quiroga, Lasa, Oliva, & Luis-Aduriz, 2014). It should be noted that many other factors contribute to consumer acceptance of modification of an existing product, for example minimising changes in texture is key for successful product reformulation.

In high sugar gel matrices, the rate limiting step of sucrose release is the diffusion of sucrose from the gel surface to the saliva (Harrison & Hills, 1996). This release is influenced by the continual generation of fresh surfaces during mastication, the rate of which is therefore dependent on the breakdown properties of the gel (Morris, 1993). The stagnant layer theory describes mass transfer of flavour compounds as being proportional to the surface area at the food-saliva interface, the mass transfer coefficient and the difference in concentration between the food and saliva (Hills & Harrison, 1995). Previous studies which correlate flavour release with perception over time from chewing gum, showed that flavour perception follows sucrose release, rather than aroma release, which has a different release profile (Davidson, Linforth, Hollowood, & Taylor, 1999), and that this was driven by cross-modal flavour interactions (Koliandris, Lee, Ferry, Hill, & Mitchell, 2008). Furthermore, diffusion rates of sucrose and resulting sweetness perception is affected by the concentration and type of hydrocolloid, this has been shown to be higher in gels with a weaker network structure (Bayarri et al., 2001, Bayarri et al., 2007, Marshall and Vaisey, 1972). Research on model gels has also shown that the rate of volatile release from model gels has a greater influence on sensory perception than maximum in-nose volatile concentration (Baek, Linforth, Blake, & Taylor, 1999). All of these combined factors should therefore be taken into account when trying to minimise changes to consumer perception after product reformation.

To the best of the authors’ knowledge, whilst a number of studies have investigated individual aroma or taste release paradigms (Tournier, Sulmont-Rossé, & Guichard, 2007), there is limited or no published work correlating both sucrose and aroma release from gel-based products with flavour perception over time. The aim was therefore to explain the impact of air inclusions on flavour release (sucrose and aroma) and temporal flavour perception in a gel based system with minimal texture changes, such that insight and knowledge could be generated for the reduction in sugar of commercial food gels.

## 2 Materials and methods

### 2.1 Preparation of gels

All ingredients were food grade and samples were prepared in a food grade environment in the Food Processing Facility, University of Nottingham. Three gels of the same volume were prepared for flavour and sensory analysis, sample N = non-aerated, sample S = slightly aerated and sample V = very aerated. 24% w/w glucose syrup and 37% w/w sucrose were dissolved in water and 10% w/w gelatine (type A, 240 bloom, mmingredients UK) and 5% w/w corn starch (Cargill UK) were added and the gelatine left to hydrate (25 °C, 5 min). The mixture was then heated to allow all of the ingredients to fully dissolve (100 °C) and prepared to a total weight of 90 g. The mixture was poured into a heated mixer at 70 °C (Thermomix, Vorwerk) and mixed at 1100 RPM for 0.5 min or 2 min to achieve different levels of aeration. In the final 10 s of mixing, 0.7% w/w citric acid, 0.3% w/w trisodium citrate and 10 g flavour (0.015% selected aroma compounds in water, or 2% IFF strawberry flavouring in water) was mixed in and the gel immediately covered to form a closed system and frozen (-80 °C) to stabilise the aerated structure. Gels were taken out of the freezer and incubated at 4 °C for 24 h before analysis and then removed from the fridge and equilibrated at 20 °C for 1 h prior to analysis. Preliminary experiments were conducted to ensure repeatability of the production method.

### 2.2 pH analysis

Sample pH was measured using a pH meter (Mettler Toledo).

### 2.3 Texture analysis

A texture profile analysis (TPA) test was carried out on the samples using a TA.TX Plus Texture Analyser (Stable Micro Systems Ltd., Surrey, UK). The samples were compressed using a 4 mm cylindrical probe according to the conditions described by Delgado and Bañón (2015), except a 0.01 N trigger force was used. As not all TPA parameters are applicable to all samples, the parameters hardness, cohesiveness and springiness were tested. These enabled calculations of chewiness by multiplying the three parameters together (Steffe, 1996).

### 2.4 X-ray computed tomography

The microstructure of the gels was analysed by X-ray μCT (Hounsfield Facility, University of Nottingham) using a Phoenix Nanotom NF180 X-ray CT System (GE Sensing & Inspection Technologies GmbH, Wunstorf, Germany), the method was modified from Yang et al. (2012). Samples were scanned in a plastic tube and consisted of 3600 projection images collected over a 360° rotation using an electron acceleration energy of 85 kV, a current of 140 μA, a detector timing of 500 ms and a scan resolution of 7 μm.

### 2.5 Image analysis

Radiograph images were reconstructed using Datos|rec software (GE Sensing & Inspection Technologies GmbH, Wunstorf, Germany) from the centre of the scanned sample to eliminate any differences at its edges. Images were imported into Image J processing software (public domain Java analysis programme, National Institute of Health, Maryland US) and cropped to a consistent size of 1000 × 1000 × 1224 px (7 × 7 × 8.568 mm).

The stack images were imported into VG StudioMAX v2.2 (Volume Graphics GmbH, Heidelberg, Germany) and the defect analysis tool was used to quantify the microstructure. Automatic surface determination was used first to segment the bubbles in the samples from the solid. Total bubble number, volume and size were determined from the software directly using the defect analysis tool. Top, front and side view images were exported from the software.

### 2.6 Sensory evaluation

The sensory study was approved by the Biosciences Ethical Committee at the University of Nottingham. A trained panel (n = 10) was recruited from the Sensory Science Centre at the University of Nottingham. Time-intensity was used to record how the attributes sweetness and overall flavour (strawberry) change over time. Texture perception of hardness and chewiness ratings on a scale of 1–10 were made with training using references, detailed in later paragraphs. Texture was rated separately to the flavour attribute measurements over time.

Panellists were asked to evaluate one attribute at a time (sweetness or overall flavour). Panellists were trained on the use of the scale and familiarised themselves with rating perceived intensity over time using a defined chewing protocol. Training reference samples were provided: for sweetness, samples were gels with high and low sugar (±50% sucrose) and the standard reference sample (100% sucrose); for overall flavour, samples were gels with high and low strawberry flavour (5% and 0.2%) and the standard reference sample (2% strawberry flavour, 100% sucrose) .

Panellists were trained on a specific chewing protocol. Panellists were asked to chew the samples every second until 5 s trained using a timer on screen, then they were asked to rest and breathe in, then repeat this cycle three times until 20 s, samples were then expectorated and measurements continued until 50 s. The study only focused on the chewing stage as preliminary experiments showed no significant differences in persistence of aroma after either spitting the sample out or swallowing the sample.

Coded samples were provided on small spoons to be placed directly in the mouth. The samples were randomised and presented to panellists in individual sensory booths, (20 °C, red lighting). Panellists were asked to rate perceived intensity of a particular attribute (sweetness or overall flavour), over a period of time using a scale from 0% to 100% (Compusense cloud, Compusense, Canada).

Release curves and extracted parameters, Imax (maximum intensity of perception of the attribute measured), Tmax (time to maximum intensity) and AUC (area under curve) were exported and presented as an average of 10 panellists (3 replicates).

For texture attributes, training reference samples (gels with high and low gelatine, ± 50% gelatine) were used for panellists to agree a scale from 1 to 10 during an initial discussion. Panellists were instructed to associate hardness with the first bite of the sample and chewiness with the perception of how chewy the gel was during chewing (Brandt et al., 1963).

### 2.7 *In vitro* and *in vivo* aroma release

The headspace above the samples was evaluated using real time aroma release by APCI-MS, this is similar to previous work by Baek et al. (1999) with slightly modified settings. For *in vitro* analysis, a model system was used using select aroma compounds. Samples were placed in a sealed jar containing 0.1 mL water (to facilitate movement when stirred) at 37 °C and stirred at 250 RPM. Aroma release was measured as the sample melted in the sealed jar, using atmospheric pressure chemical ionisation – mass spectrometry (APCI-MS). Selected ion monitoring analysis was used to measure the aroma compounds ethyl acetate, hexanal, ethyl butyrate, octanal, ethyl hexanoate and decanal (protonated ions 89, 101, 117, 129, 145, and 157 *m*/*z*, respectively). Aroma release rates were calculated by taking the linear portion of the release curve and normalising to the maximum for all gel concentrations. MassLynx software (Micromass, Manchester, UK) was used to compile the data for individual compounds, CDC (University of Nottingham in house software) was used to extract the data before analysis. The *in vitro* analysis aimed to investigate whether different physicochemical properties of the aroma compounds affected their release from the matrices with variable levels of air inclusions, hence a series of linear esters and aldehydes were used.

For *in vivo* analysis, a commercial flavour was used rather than select aroma compounds for the *in vitro* experiments, as this offered a more familiar and distinct flavour profile during sensory analysis. It was easier for panellists to easily recognise the flavour of strawberry and allowed the correlation of aroma release to flavour perception. Real time aroma release was measured using APCI-MS as the product was chewed by the same trained panellists (n = 10). The predominant aroma compounds from the commercial flavour were tracked as previously described (ethyl butyrate, ethyl isovalerate, ethyl hexanoate and isoamyl butyrate, protonated ions 117, 131, 145 and 155 *m*/*z*).

### 2.8 Sucrose release

A swabbing and sucrose extraction protocol was used to measure sucrose release from the samples during chewing (Davidson et al., 1999). Preliminary experiments were conducted to develop a protocol to prevent particles of gel getting onto the swabs during the chewing stage. This involved placing samples in a piece of cheesecloth thereby ensuring that only the liquid gel and saliva was swabbed and not larger gel particles as fragmented gel pieces during the chewing stages were shown to interfere with sucrose diffusion to the taste buds (Kohyama, Hayakawa, Kazami, & Nishinari, 2016), and also prevent panellists swabbing gel pieces resulting in higher concentrations of sucrose extracted than actually present in the saliva.

The same trained panellists followed the chewing protocol for chewing the gels and the centre of the tongue was swabbed in a zig-zag motion at time points 0, 5, 10, 15, 20, 30, 40 and 50 s. Sucrose was extracted from the swabs using 3 mL 50:50 H_2_O:MeOH (Fisher Scientific, UK) solvent. After vortexing, the solution was centrifuged at 4860 RPM for 30 min, and 1 mL liquid was taken from the supernatant for liquid chromatography mass spectrometry (LC-MS) analysis. The same LC-MS settings, column and solvent described in Su, Brunet, Festring, Ayed, Foster, and Fisk (2021) were used in this study. The concentration of sucrose in the saliva was calculated using a sucrose standard curve from 37.5 to 500 mg / mL. The concentration of sucrose in the saliva was calculated using a sucrose standard curve from 37.5 to 500 mg / mL.

### 2.9 Statistical analysis

Analysis of variance (ANOVA) tests and Tukey’s HSD test were used to determine statistical differences between the samples (α = 0.05, p-value < 0.05). Two factor ANOVA was used to understand variation between panellists, sample, and panellist-sample interaction. Variation between panellists was observed (data in supplementary material) therefore, panellists were treated as individual blocks, and replicates were included in the blocks for ANOVA analysis to account for individual variation and understand differences between samples rather than differences between panellists. Partial least squares (PLS) analysis was used to identify factors that significantly correlated with each other. All statistical analysis was carried out using XLSTAT (Addinsoft, NY, USA), and Design Expert v.11 (Stat-Ease, Minneaopolis).

## 3 Results and discussion

### 3.1 Gel characteristics and structure

**Fig. 1:**
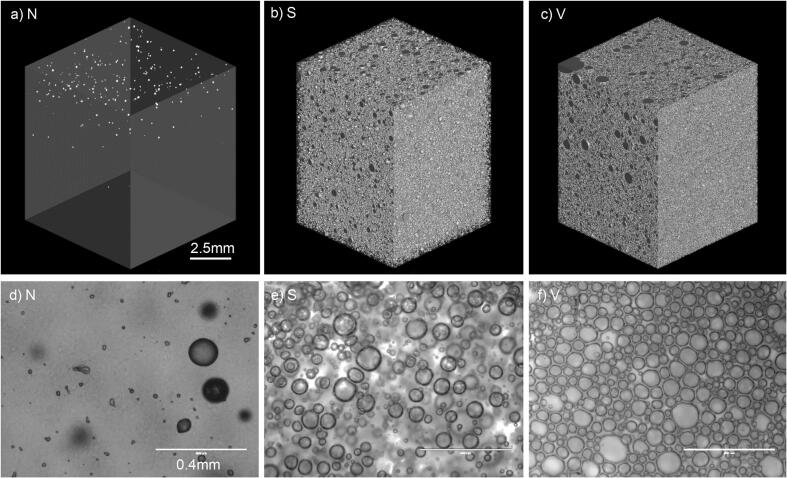
Air inclusion distribution in standard non-aerated sample, N (gel density 1.3 g / mL), slightly aerated sample, S (gel density 1.0 g / mL) and very aerated sample, V (gel density 0.8 g / mL). X-ray computed tomography (a-c) and light microscopy analysis (d-f).

Two gels with different densities were developed by aeration, these had a 23% (sample S) and 38% (sample V) reduction in density compared to the non-aerated gel (sample N) (Table 1). Since the method of producing the gels involved boiling the solution, air inclusions were present in the non-aerated gel (Fig. 1 a,d), however these were present at a significantly smaller volume fraction than the aerated gels (P < 0.05). Increased aeration did not significantly impact the mean bubble diameter (Table 1) despite it being more saturated with bubbles than the slightly aerated gel (Fig. 1 e,f). There was no significant effect on pH (P > 0.05). The mean bubble diameter (0.05–0.06 mm) for both aerated gels was in the range described as ‘microaeration’ (Haedelt et al., 2007) since the bubbles cannot be detected with the human eye. Air inclusions were distributed uniformly throughout the gel at both levels of aeration (Fig. 1 b,c),and resulted in a significant increase in overall surface area of the aerated gels.

**Table 1:** Gel characteristics ± SD analysed using texture analysis, pH meter, x-ray computed tomography and sensory evaluation. Sample N = non-aerated, S = slightly aerated, V = very aerated. Significant differences between samples are indicated by different letters in superscript.

Sample	N	S	V
Gel density (g / mL)	1.3^a^ ± 0.01	1.0^b^ ± 0.03	0.8^c^ ± 0.01
Mean air inclusion diameter (mm)	0.06^a^ ± 0.06	0.06^a^ ± 0.04	0.05^a^ ± 0.05
Hardness (N)	1.02^a^ ± 0.04	0.93^b^ ± 0.03	0.61^c^ ± 0.04
Hardness sensory rating (1–10)	4.25^a^ ± 0.65	2.82^b^ ± 0.37	1.7^c^ ± 0.41
Chewiness (N mm)	0.89^a^ ± 0.03	0.84^a^ ± 0.05	0.54^b^ ± 0.02
Chewiness sensory rating (1–10)	3.30^a^ ± 0.46	3.10^a^ ± 0.59	1.72^b^ ± 0.33
pH	3.92^a^ ± 0.02	3.91^a^ ± 0.02	3.90^a^ ± 0.02

Increased aeration significantly decreased the hardness of the gels, this was measured as perception of texture during the first bite (Table 1). This was significant for both the sensory attribute, hardness (P < 0.05) and instrumentally measured hardness by texture analysis (P < 0.05). There was no significant difference in chewiness perception between the non-aerated gel and the slightly aerated gel, this was also shown using the instrumentally measured chewiness by texture analysis (P < 0.05). These results highlight how sensory evaluation of texture correlates well with the TPA method, which has also been shown previously by Clark (2006). Hardness and chewiness of gels has been shown to affect sweetness perception and have be used to predict sweetness (Marshall & Vaisey, 1972). Despite the slightly aerated gel (S) being filled with air bubbles (Fig. 1 b), this reduction in density did not affect the perceived chewiness of the gel when compared to the non-aerated gel (N). Aerating the gel further did affect chewiness perception, the very aerated gel (V) was significantly less chewy when compared to the non-aerated (N) and slightly aerated (S) samples (P < 0.05). These findings suggest that whilst air inclusions have a direct impact on perception at first bite (hardness), for texture perception throughout chewing (chewiness), a critical amount of air inclusions are required to impact perception and instrumentally measured chewiness. Therefore, low levels of air inclusions could be included with negligible impacts on perceived chewiness.

Minimising texture perception changes is important for reformulation using this strategy since texture changes have been a limitation of this strategy discussed in previous work (Chiu et al., 2015, Zúñiga and Aguilera, 2008). Although texture differences have been shown to be minimised, there were still differences in the colour of the product, as sample S was slightly more opaque than N, and sample V was opaque and solid white in colour as a result of increased light scattering. Similar differences were described by Chiu et al. (2015) in soft gels, and these factors should be considered when aerating products. Although, Marshall and Vaisey (1972) showed that there was no clear relationship between colour differences of gels and sweetness perception.

### 3.2 *In vitro* aroma release

**Fig. 2:**
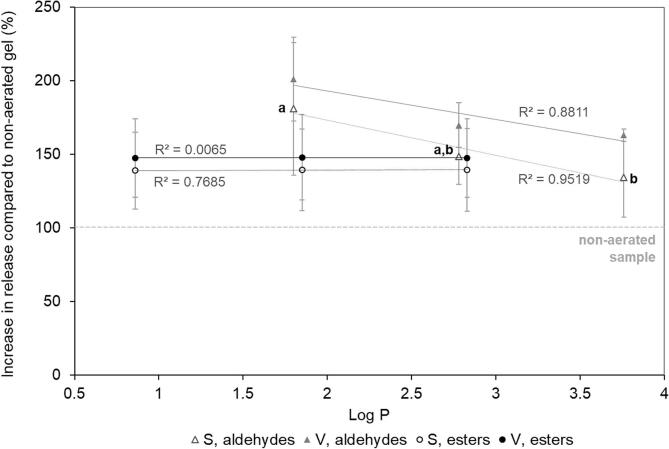
Effect of hydrophobicity (Log P) of aroma compound on release rate from aerated gels compared to non-aerated (dashed line, 100%). Slightly aerated sample, S (gel density 1.0 g / mL) are show as empty circles and triangles, and very aerated sample, V (gel density 0.8 g / mL gel) are shown as filled circles and triangles. Different letters show significant differences in aroma release, labelled only on samples with statistical differences. Aldehydes: hexanal, octanal, decanal; esters: ethyl acetate, ethyl butyrate, ethyl hexanoate. Trend lines show correlations between effect of aeration and hydrophobicity on release.

Gels were stirred and melted at physiological temperatures and the release of aroma evaluated using ACPI-MS. A steady release of aroma was observed *in vitro* followed by a plateau, which correlated with when the gels had fully melted and aroma partitioning was at equilibrium. Release rates were calculated from the initial linear section of the release curve, the relative increase in release rate compared to the non-aerated gel is shown in Fig. 2 for each aroma compound. Release rates of all aroma compounds from both aerated gels was significantly greater than the non-aerated gel, however aerating more did not have a significant impact on release rate compared to just aerating slightly (P > 0.05). This suggests that there may be an optimum level of aeration, as aerating more will reduce the density of the product further, but with significant textural changes and no benefit in aroma release rates compared to lower levels of aeration. Therefore, low levels of aeration appear to increase aroma release rates whilst minimising texture changes.

Release rate of esters was independent of their hydrophobicity, since there was no significant difference between the esters and between the different gels. However, there seemed to be a slight effect of hydrophobicity on the release of aldehydes, as the relative increase in release rate decreased with increasing hydrophobicity. Specifically, there was significantly less (P < 0.05) of an impact of aeration on the release of decanal compared to hexanal (Fig. 2). Since the impact of aeration was different for different groups of aroma compounds, by aerating a sample, the balance of aroma in the final product may be affected. Although aroma release rate was increased, aroma concentrations may need to be adjusted to maintain the same balance of aroma in the end product, in a similar way to that previously shown for fat reduction (Malone et al., 2000, Malone et al., 2003, Yang et al., 2011).

Aeration creates a hydrophobic phase in a predominantly hydrophilic gel matrix, hydrophobic aroma volatiles may therefore migrate into the hydrophobic gas voids. Further studies could investigate how volatile compounds distribute when this additional gas phase is created and whether they partition to a greater or lesser extent in the hydrophilic or hydrophobic regions of the gel and if they accumulate in the air inclusions.

### 3.3 Flavour perception over time

**Fig. 3:**
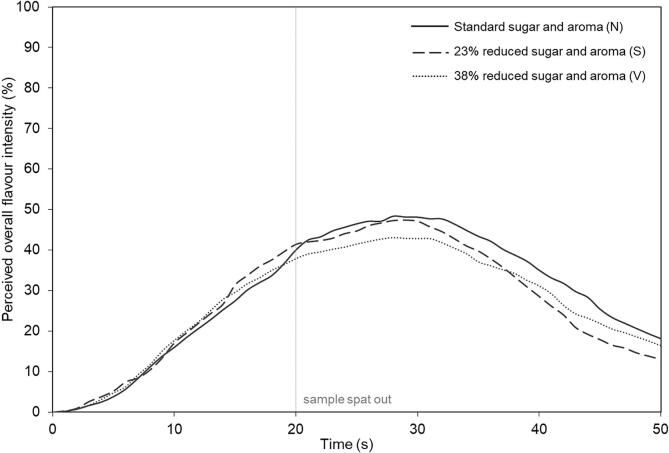
Effect of aerating confectionery gels (samples S and V), and reducing density of gel sweets on their perceived flavour intensity over time compared to a standard non-aerated gel (N). Non-aerated sample, N (gel density 1.3 g / mL), slightly aerated sample, S (gel density 1.0 g / mL), very aerated sample, V (gel density 0.8 g / mL).

The three gels were evaluated for the attribute overall flavour using time-intensity by a trained sensory panel (n = 10). No significant difference was observed in the perception of overall strawberry flavour during consumption (Tmax, Imax, P > 0.05, Fig. 3, supplementary material). This was despite a 23% and 38% reduction in both aroma and sugar in the gel on a weight basis and a significant reduction in density. This result is in agreement with static measurements of saltiness perception in previous studies (Chiu et al., 2015) and sweetness perception (Goh et al., 2010), which showed that taste perception is maintained when the concentration of tastants is reduced through addition of air inclusions.

Initial flavour perception was also similar for all three gels, this can be seen in the initial development in flavour intensity in Fig. 3. This is a key phase in consumer acceptance of products and suggests that that flavour similarity might be possible for consumers, with reduced sugar products containing air inclusions.

No significant differences (P > 0.05) were observed in the interaction between panellist and sample (supplementary material), indicating similar use of scale and rating of the sample in a similar way. This gives confidence in panel performance.

These results can be explained by considering aroma release and tastant release. Partial least squares (PLS) regression analysis was carried out on all of the sensory and analytical parameters to identify factors that significantly correlated with each other (supplementary material). Sample V correlated with greater sucrose release and faster perception of flavour. Sample S and N correlated with a high chewiness and hardness, which is projected in a group with maximum intensity of aroma release of the four compounds, and this is also expected since these samples have a higher aroma content.

### 3.4 *In vivo* aroma release

**Fig. 4:**
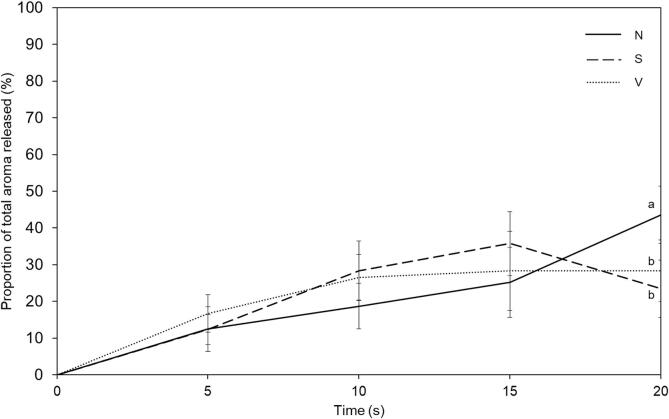
Proportion of ethyl butyrate released during the time course of chewing a non-aerated confectionery gel (N), compared to aerated confectionery gels (S and V), Error bars show the 95% confidence interval at each time point calculated from pooled standard deviation, different letters indicate significant differences between the samples and are labelled only on significantly different samples. Non-aerated sample, N (gel density 1.3 g / mL), slightly aerated sample, S (gel density 1.0 g / mL), very aerated sample, V (gel density 0.8 g / mL).

Aroma release in real time was measured using APCI-MS with the same panellists that evaluated perceived flavour intensity over time. Since the controlled chewing protocol involved four distinct sets of chews before expectorating the sample, total aroma release during each set of chews was calculated to understand differences in aroma release *in vivo*. A greater proportion of the total aroma was released during the first half (10 s) of chewing for the aerated samples (Fig. 4). This contrasts with the non-aerated sample N where a significantly greater proportion (44%) of the total ethyl butyrate was released during the last set of chews (P < 0.05). Only ethyl butyrate was shown, as an example of the four compounds measured, as the release patterns of all four compounds measured was similar. This reinforces the fact that by adding air inclusions into the matrix, a faster release of aroma can be achieved both *in vitro* (Fig. 2) in equilibrium conditions and *in vivo* (Fig. 4) during chewing of the sample. This also supports the stagnant film theory proposed by Hills & Harrison (1995), whereby an increase in surface area for exchange at the food-saliva interface, results in increased rate of diffusion of aroma compounds and hence faster release.

As the gels have the same volume, it was expected that there would be a reduction in the maximum concentration of aroma with increased levels of aeration due to a significant reduction in the total material consumed (supplementary material). Yet despite this decrease in weight, the same overall flavour was perceived (Fig. 3). These results further support previous results on model gels (Baek et al., 1999) which showed that sensory perception is affected by rate of release, rather than maximum in-nose concentration.

The behaviour of all esters measured was similar, in that there was a decrease in maximum intensity of release in gels with lower density through aeration which is in agreement with *in vitro* release data that esters behave similarly in matrices with air inclusions (Fig. 2). The mechanism by which air inclusions enhance the release of aroma compounds such as esters is more likely a surface area effect, where an increased surface area results in faster mass transfer of aroma at the interface (Hills & Harrison, 1995).

However, conclusions on the mechanisms cannot be fully drawn as only four compounds in the strawberry flavour were measured using APCI-MS due to the low concentration of some of the aroma compounds. It is important to consider that other aroma compounds present in the commercial flavours, (e.g. *cis*-3-hexenol and γ-decalactone), will contain a wider range of functional groups and therefore will have different physicochemical properties, these compounds may therefore respond differently to the addition of air inclusions within the matrix. The reduction in Imax of esters *in vivo* as a result of aeration, may be a minimal change compared to other compounds. These other changes may be important in affecting overall flavour perception, and the reason that no significant difference in flavour perception was observed. Rebalancing flavours after reformulating with aeration can be easily achieved if the behaviour of a larger range of aroma volatiles of varying hydrophobicity and functional groups is understood, as predictive models can be built. In previous studies, an increase in flavour compounds was required to balance overall flavour perception as a result of reducing sucrose in confectionery gels (Riedel, Böhme, & Rohm, 2015). Ultimately in our study, due to the successful redesign of the product structure (air inclusions), sensory perception was unaffected even with a reduction in total weight of sugar and aroma compounds.

### 3.5 Sucrose release

**Fig. 5:**
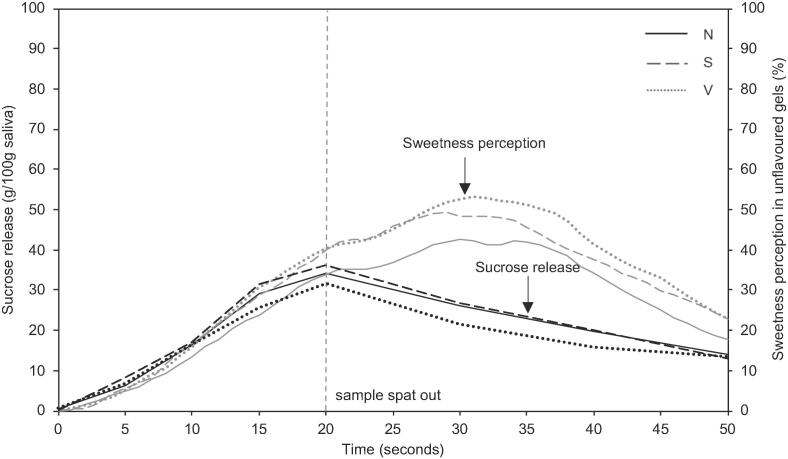
Sucrose release (black) and sweetness perception (grey) from aerated (samples S, V) and non-aerated (N) standard confectionery gels. Non-aerated sample, N (gel density 1.3 g / mL), slightly aerated sample, S (gel density 1.0 g / mL), very aerated sample, V (gel density 0.8 g / mL).

The release of sucrose over time from the three samples was similar despite a 23% and 38% reduction in density and sucrose in the aerated gels (Fig. 5). No significant difference was observed between the gels in terms of maximum concentration of release of sucrose (supplementary material). By increasing the surface area of each fragment of chewed gel through aeration, and thereby increasing the rate of diffusion of sucrose (Harrison & Hills, 1996), a greater proportion of the total sucrose was released from the aerated gels. The release curves of sucrose are similar at each time point and no decrease in sweetness perception was observed (Fig. 5 and supplementary material). When the aerated gels are broken down compared to the non-aerated gel, the fragments of gel have a higher surface area which is in contact with the saliva phase. The multi-fragmentation theory in the context of cube shaped gels, already predicts an exponential increase in surface area (Hills & Harrison, 1995). With the repeated generation of fresh surfaces resulting from each chew (Morris, 1993) being greater due to aeration, ultimately, there is a much larger area at the food-saliva interface for sucrose to diffuse out of the gel matrix into the saliva. Also, as previous studies have shown, sucrose diffusion is faster from gels with a weaker network structure (Bayarri et al., 2001).

Panellists were asked to rate perceived sweetness intensity over time in gels with no aroma and to focus on only the parameter sweetness. A significant difference in Imax was observed between the aerated gels and the non-aerated gel (P < 0.05), despite there being no difference in sucrose release over time. No significant difference was observed in Tmax (P > 0.05, supplementary material). Similar results, which showed no change in Tmax were previously reported for sweetness perception of gels with different textures (Bayarri et al., 2007). There was a delay between maximum sensory perception of sweetness and maximum sucrose release (Fig. 5) rather than following sucrose release reported by Davidson et al. (1999). This may be due to the different matrix and a direct result of mouth coating, whereby when the gel melts in the mouth it forms a film on the tongue such that when the product is swallowed, a layer remains. This may have prolonged the sweetness perception and resulted in panellists perceiving sweetness after the point of spitting out the sample. Similar findings were reported by Kohyama et al. (2016) that maximum sweetness was after the oral processing time and the authors also suggested that sucrose from gels remained in the mouth.

## 4 Conclusions

By slightly aerating a gummy confectionery gel, whilst maintaining a fixed volume, a reduction in gel density can be achieved without affecting chewiness, a key texture attribute of gummy confectioneries. Air inclusions increased the surface area formed during chewing which enabled a faster diffusion of aroma and sugar from the food matrix into the saliva, this compensated for the reduction in sugar and aroma in the product. Ultimately, the same overall perception of flavour was achieved for aerated gels that had 23% and 38% reduced levels of aroma and sucrose when compared to the non-aerated products. Although flavour perception remains unchanged, the effect of air inclusions varied for individual groups of compounds and therefore more complex aroma mixtures may need to be rebalanced. This study has highlighted the importance of investigating a range of aroma volatiles with different properties and further studies should aim to understand release of compounds of other functional groups, to be able to easily rebalance the aroma profile of complex mixtures.

## CRediT authorship contribution statement

**Katy Su:** Investigation, Conceptualization, Methodology, Validation, Formal analysis, Visualization, Writing - original draft, Writing - review & editing. **Daniel Festring:** Supervision, Writing - review & editing. **Charfedinne Ayed:** Formal analysis, Supervision, Writing - review & editing. **Qian Yang:** Methodology, Supervision, Writing - review & editing. **Craig J. Sturrock:** Methodology, Resources, Investigation, Writing - review & editing. **Robert Linforth:** Formal analysis, Writing - review & editing. **Tim Foster:** Supervision, Writing - review & editing. **Ian Fisk:** Funding acquisition, Conceptualization, Supervision, Visualization, Writing - review & editing.

## Declaration of Competing Interest

The authors declare that they have no known competing financial interests or personal relationships that could have appeared to influence the work reported in this paper.
