# Supplementary material for: Reducing sugar and aroma in a confectionery gel without compromising flavour through addition of air inclusions
Source: Food Chem. 2021 Aug 30;354:129579. doi: 10.1016/j.foodchem.2021.129579 (PMC8091041; doi:10.1016/j.foodchem.2021.129579)
Supplement: Supplementary Data 1 [file mmc1.docx]

**Supplementary Material**

Reducing aroma and sugar in a confectionery gel without compromising flavour through addition of air inclusions

S1, Table 1 - sensory and analytical parameters ± pooled standard deviation of 3 replicates for 10 individuals. Different letters indicate significant differences within each row of data, analysed using ANOVA with panellists grouped as individual blocks to examine significant differences between samples rather than differences between panellists. Effect of block was analysed, parameters with no significant effect of block are indicated by ^*^.

|  | N | S | V |
| --- | --- | --- | --- |
| Aroma (M+1) |  | Imax (ppb) |  |
| Ethyl butyrate (117) | 3291^a^ ± 641 | 2669^b^ ± 432 | 1941^c^ ± 313 |
| Ethyl isovalerate (131) | 6519^a^ ± 1370 | 4347^b^ ± 1382 | 2463^c^ ± 563 |
| Ethyl hexanoate (145) | 1394^a^ ± 427 | 921^b^ ± 457 | 488^c^ ± 107 |
| Isoamyl butyrate (159) | 162^a^ ± 54 | 87^b^ ± 44 | 44^c^ ± 10 |
|  |  |  |  |
| Sucrose |  |  |  |
| Imax (g/100g saliva) | 44.1^a^ | 45.1^a^ | 36.0^a^ |
| AUC sucrose | 1093.2^a^ | 1070.4^a^ | 920.6^a^ |
|  |  |  |  |
| Sensory perception sweetness |  |  |  |
| Imax | 46^a^ ± 11 | 56^b^ ± 8 | 56^b^ ± 8 |
| Tmax (s) | 29^a^ ± 4 | 29^a^ ± 4 | 29^a^ ± 3 |
|  |  |  |  |
| Sensory perception overall flavour |  |  |  |
| Imax | 55^a*^ ± 12 | 54^a*^ ± 11 | 50^a*^ ± 7 |
| Tmax (s) | 29^a^ ± 2 | 27^a^ ± 4 | 28^a^ ± 4 |
|  |  |  |  |

S1, Table 2 – overall panel performance assessed by ANOVA for sensory analysis, and interpanel variation for aroma and sucrose analysis. Values in bold shows significant effect of panellist on result observed in S1 Table 1, sample or panellist * sample interaction (p<0.05).

|  | p-value | | |
| --- | --- | --- | --- |
|  | Panellist | Sample | Interaction |
| Aroma (M+1) |  |  |  |
| Ethyl butyrate (117) | **<0.001** | **<0.001** | **0.01** |
| Ethyl isovalerate (131) | **<0.001** | **<0.001** | 0.46 |
| Ethyl hexanoate (145) | **<0.001** | **<0.001** | 0.58 |
| Isoamyl butyrate (159) | **<0.001** | **<0.001** | 0.38 |
| Sucrose |  |  |  |
| Imax (g/100g saliva) | **0.032** | 0.51 | 0.93 |
| AUC sucrose | **<0.001** | 0.64 | 0.28 |
|  |  |  |  |
| Sensory perception sweetness |  |  |  |
| Imax | **<0.001** | 0.14 | 0.18 |
| Tmax (s) | **<0.001** | 0.95 | 0.20 |
|  |  |  |  |
| Sensory perception overall flavour |  |  |  |
| Imax | **0.048** | 0.95 | 0.81 |
| Tmax (s) | **<0.001** | 0.53 | 0.88 |
|  |  |  |  |

S1, Figure 1 – partial least squares regression analysis of all sensory and analytical parameters.

Partial least squares (PLS) regression analysis was carried out on all of the sensory and analytical parameters to identify factors that significantly correlated with each other. Three main groups of factors were identified:

1. Total aroma release (calculated from and represented by the area under curve (AUC 117, 131, 145 and 159) is highly projected in axis 1.
2. The second group of factors is projected on negative axis 1, including the factors ‘Tmax Perception’ ‘Imax Sucrose’ ‘AUC Sucrose’
3. Anti-correlated with these groups, a group of factors projected on negative axis 2 include ‘Imax 89, 117, 145 and 175’ as well as ‘Hardness’ and ‘Chewiness’.

Projections of panellists and samples N, S and V show the correlation and anti-correlation to certain groups of factors.

PLS analysis also highlights the effect of panellists on the differences in perception and release, and individual variation in chewing and processing was also observed as the majority of panellists were distributed around negative axis 1 and 2, however panellist 7 was spread out from the general cluster. Sample N and S mostly correlates with group of factors (group 3) projected in negative axis 2 and sample V correlates with the group of factors (group 2) projected in negative axis 1.
